# Calcitonin-responsive adenylate cyclase in a calcitonin-producing human cancer cell line.

**DOI:** 10.1038/bjc.1977.119

**Published:** 1977-06

**Authors:** N. H. Hunt, M. Ellison, J. C. Underwood, T. J. Martin

## Abstract

A calcitonin-responsive adenylate cyclase has been found in a cell line of a poorly differentiated bronchial carcinoma (BEN cells). The cells have previously been shown to secrete an immunoreactive form of calcitonin in culture. Salmon calcitonin (SCT), porcine calcitonin (PCT) and human calcitonin (CT-M) all stimulated adenylate cyclase activity in particulate preparations. CT-M sulphoxide had little effect. The concentrations of the calcitonins required for half the maximum activation of adenylate cyclase were 6-8, 18 and 90 nm respectively. SCT (30pm) and CT-M (60 pm) increased the intracellular concentration of cyclic AMP from 11-2+/-0-2 (s.e.) to 18-2+/-0-2 and 16-7+/-0-2 respectively over a 2-5-min period. SCT (labelled with 125I) bound to particulate preparations of Ben cells, and competition for binding occurred with unlabelled SCT and CT-M. The concentration of SCT required for half the maximum inhibition of [125I]SCT binding was 11 nm. CT-M sulphoxide inhibited only at high concentration (3 micron). The characteristics of the adenylate cyclase response to SCT did not change over the period between cell adhesion (after subculture) and confluence. However, pre-incubation of cells for 4 h with SCT (150 nm) abolished the subsequent adenylate cyclase response of particulate preparations to further hormone. The practical difficulties encountered in purifying and quantifying the large-mol.-wt. form of CT-M secreted by BEN cells has precluded direct investigation of the potential relationship between hormone secretion and the occurrence of the calcitonin receptor. This relationship is discussed in terms of its possible biological significance.


*Br. J. Cancer* (1977) **35**, 777.

### CALCITONIN-RESPONSIVE ADENYLATE CYCLASE IN A CALCITONIN-PRODUCING HUMAN CANCER CELL LINE

N. H. HUNT,* M. ELLISON,† J. C. E. UNDERWOOD‡ AND T. J. MARTIN*

*From the *Depts. of Chemical Pathology and ‡Pathology, Sheffield University Medical School, Beech Hill Road, Sheffield, S10 2RX; and †the Unit for Human Cancer Biology, Ludwig Institute for Cancer Research (London Branch) in conjunction with the Royal Mardsen Hospital, London*

Received 15 November 1976 Accepted 25 January 1977

**Summary.—A calcitonin-responsive adenylate cyclase has been found in a cell line of a poorly differentiated bronchial carcinoma (BEN cells). The cells have previously been shown to secrete an immunoreactive form of calcitonin in culture. Salmon calcitonin (SCT), porcine calcitonin (PCT) and human calcitonin (CT-M) all stimulated adenylate cyclase activity in particulate preparations. CT-M sulphoxide had little effect. The concentrations of the calcitonins required for half the maximum activation of adenylate cyclase were 6·8, 18 and 90 nM respectively. SCT (30 pM) and CT-M (60 pM) increased the intracellular concentration of cyclic AMP from 11·2±0·2 (s.e.) to 18·2 ± 0·2 and 16·7 ± 0·2 respectively over a 2·5-min period.**

**SCT (labelled with $^{125}$I) bound to particulate preparations of BEN cells, and competition for binding occurred with unlabelled SCT and CT-M. The concentration of SCT required for half the maximum inhibition of [$^{125}$I]SCT binding was 11 nM. CT-M sulphoxide inhibited only at high concentration (3μM).**

**The characteristics of the adenylate cyclase response to SCT did not change over the period between cell adhesion (after subculture) and confluence. However, preincubation of cells for 4 h with SCT (150 nM) abolished the subsequent adenylate cyclase response of particulate preparations to further hormone. The practical difficulties encountered in purifying and quantifying the large-mol.-wt. form of CT-M secreted by BEN cells has precluded direct investigation of the potential relationship between hormone secretion and the occurrence of the calcitonin receptor. This relationship is discussed in terms of its possible biological significance.**

THE development of neoplasia in animal tissues is associated with striking changes in cell function. Release of "foetal reversion products", such as carcinoembryonic antigen (CEA) and α-foetoprotein (Neville and Cooper, 1976) and ectopic hormone production (Rees and Ratcliffe, 1974) are common examples. Other phenomena which have been observed are changes in the number or type of hormone receptors at the cell surface (Schorr *et al.*, 1971; Christoffersen *et al.*, 1972; Christoffersen *et al.*, 1974; Boyd, Louis and Martin, 1974; Christoffersen and Berg, 1975) and alterations in the metabolism of cyclic nucleotides (Clark, Morris and Weber, 1973; Minton, Wisenbaugh and Matthews, 1974; Ryan and Heidrick, 1974; DeRubertis, Chayoth and Field, 1976). In reviewing evidence in the latter field, it was apparent that intracellular cyclic adenosine 3′,5′-monophosphate (cyclic AMP) levels could not be regarded as having a simple negative correlation with growth, particularly in view of the fact that some tumours *in vivo* had high levels of cyclic AMP (Minton *et al.*, 1974) and of adenylate cyclase (Martin *et al.*, 1976). This led to the view that the regulation of intracellular cyclic AMP levels might be less important for cell growth than for the expression of

Reprint requests to Professor T. J. Martin.

certain metabolic functions of cancer cells.

The present experiments arose out of studies of hormone-responsive adenylate cyclase in cancer cells possessing the particular function of "inappropriate" hormone production (Martin *et al.*, 1976). A human cancer cell line which continuously secreted a large-mol.-wt form of human calcitonin (Ellison *et al.*, 1975, 1976*a*; Coombes *et al.*, 1976, 1977) was found to contain an adenylate cyclase which responded to biologically active calcitonin. The present report describes some properties of the hormone-responsive adenylate cyclase and demonstrates specific binding of labelled calcitonin to particulate fractions of the cells. Some evidence is also presented bearing upon the biological activity of the secreted calcitonin and the relationship to the "feedback" control which is apparent in culture.

### METHODS

*Cell culture.*—An epithelial cell line (BEN) was established from a poorly differentiated epidermoid bronchial carcinoma, as described previously (Ellison *et al.*, 1975). Cells were cultured in 45% TC199 HEPES buffered medium (Biocult) plus 45% Dulbecco's Eagle's bicarbonate-buffered medium, plus 10% outdated human plasma in 25-cm$^2$ plastic Falcon flasks. 100 $\mu$g/ml kanamycin and 2·5 $\mu$g/ml amphotericin B were added to the medium. Cultures were gassed (5% $CO_2$ in air) and maintained at 37°C. Cells were subcultured each week. They have continued to secrete CEA and calcitonin since the earlier report (Ellison *et al.*, 1975).

*Hormones.*—Synthetic salmon calcitonin (SCT) lot No. K600128C-1, was a gift from Dr J. W. Bastian, Armour Pharmaceutical Co., Kankakee, Illinois, and human calcitonin (CT-M) and CT-M sulphoxide were gifts from Dr W. Rittel, CIBA, Basle, Switzerland. Human growth hormone (Commonwealth Serum Laboratories, Parkville, Australia) was clinical-grade material, and ovine prolactin, also partially purified, was a gift from Dr C. J. Robinson, Dept. of Physiology, University of Newcastle upon Tyne. Prostaglandins were a gift from Dr John Pike, Upjohn Co., Kalamazoo, Michigan, and pentagastrin was obtained from I.C.I. Sources of parathyroid hormone, histamine, vasopressin, glucagon and isoprenaline were as listed previously (Martin *et al.*, 1976).

*Adenylate cyclase activity.*—Assays were carried out when cell cultures were at or near confluence, unless otherwise indicated. Medium was decanted from the culture flasks and the cells rinsed twice with 5 ml 0·154M NaCl. Cells were scraped from the surface with a plastic "policeman" and homogenized in an all-glass, hand-held homogenizer (6 strokes) in the buffer used for the enzyme assay (25 mM Tris HCl, pH 7·8, 4·5 mM $Mg^{2+}$, 30 mM $K^+$). After centrifugation (2200 $g$ for 10 min) the particulate preparation was resuspended for assay. The adenylate cyclase assay system has been described previously (Hunt *et al.*, 1976). Briefly, [$\alpha$-$^{32}$P]ATP was incubated with particulate preparations in a volume of 100 $\mu$l for 20 min at 30°C in the presence of the necessary ions, an ATP-regenerating system and 1 mM unlabelled cyclic AMP to inhibit breakdown of labelled cyclic AMP. The reaction was stopped by boiling, after adding an excess of unlabelled ATP, and the [$^{32}$P] cyclic AMP was purified by sequential chromatography on columns of Dowex and alumina (Salomon, Londos and Rodbell, 1974). [$^3$H]cyclic AMP added at the column stage was used to monitor recoveries, which were 75–85%. Protein concentrations were measured by the method of Hartree (1972).

*Cyclic AMP measurement.*—Replicate cultures were established in plastic Leighton-type tubes. When the cultures had grown to near confluence (approximately $3 \times 10^6$ cells per tube) media were changed and a 30-min equilibration period allowed, in medium containing 0·1% bovine serum albumin (Armour) and no plasma. To this medium was added either hormone in 20 $\mu$l 10 mM acetic acid or acetic acid alone. Ten min later the medium was decanted, the cells were quickly rinsed twice with 3 ml cold 0·154M saline, and 1 ml 50 mM $NH_4OH$ was added to remove the cells. After boiling for 5 min, the residue was freeze-dried and reconstituted for assay by the protein-binding method of Brown *et al.* (1971). The overall procedure was validated by (i) adding unlabelled cyclic AMP to cell cultures and showing that recovery was quantitive, and (ii) establishing parallelism between serially diluted samples and assay standards.

*Calcitonin binding*.—Synthetic salmon calcitonin was labelled with $^{125}I$ by the method of Hunter and Greenwood (1962), using 2 μg of chloramine T as oxidant. In the case of SCT this has been shown to produce biologically active labelled hormone (Marx, Fedak and Aurbach, 1972; Sraer and Ardaillou, 1973). After purification using Quso G32 (Yalow and Berson, 1966) the [$^{125}I$]SCT was used for binding studies. Specific activity was consistently between 80 and 120 μCi/μg. Incubations were carried out at 25°C in plastic tubes (12 × 75 mm) in a total volume of 200 μl, consisting of 50 mM Tris-HCl, pH 7·4, 2 g/100 ml bovine serum albumin, 1 mM disodium EDTA. Tubes contained 20,000 ct/min of [$^{125}I$]SCT and varying amounts of unlabelled SCT. After preliminary experiments showed that binding was maximal at 10 min, and began to decline slowly at 20 min, incubation times of 15 min were used. At the end of this period, 100 μl samples were removed from incubation mixtures and layered on top of 200 μl of 10 g/100 ml sucrose in polypropylene tubes and centrifuged at 10,000 *g* for 2 min in a Beckman Microfuge. After aspirating the supernatant, the tips of the tubes were cut off and counted in a Packard Auto Gamma counter.

### RESULTS

When a number of hormones were tested at high concentrations, the adenylate cyclase of the BEN cell membranes was activated by isoprenaline, prolactin, human growth hormone, and SCT (Table I). The greatest stimulation was obtained with SCT and this was always of greater magnitude than the effect of fluoride. The effects of prolactin and growth hormone were small but reproducible, and were seen only at high concentrations. Isoprenaline also consistently caused a minor increase in adenylate cyclase activity, but this was not investigated in detail. When the calcitonin response was examined further, calcitonins of three species were found to produce dose-dependent effects on adenylate cyclase at low hormone concentrations (Fig. 1). Half-maximal activation of adenylate cyclase by porcine, human and salmon calcitonins was produced by concentrations of 18 nM, 90 nM and about 7 nM respectively. The sulphoxide form of CT-M, which has little or no calcium-lowering activity in the rat (Neher *et al.*, 1968), produced a small activation of adenylate cyclase at a concentration of 600 nM.

When intact cells were tested, both SCT and CT-M increased the cellular levels of cyclic AMP over a 2·5-min period. Control cells contained 11·2 ± 0·2 (s.e.) pmol cyclic AMP/tube, whereas SCT (30 pM) increased the level to 18·2 ± 0·2 pmol ($P < 0·001$) and CT-M (60 pM) increased it to 16·7 ± 0·2 pmol ($P < 0·001$).

TABLE I.—*Effect of Hormones on Adenylate Cyclase Activity in Particulate Preparations of BEN Cells. $PGE_1$ and $PGE_2$, Prostaglandins $E_1$ and $E_2$; PTH, Parathyroid Hormone; HGH, Human Growth Hormone; SCT, Salmon Calcitonin. Values are Means ± s.e. from 3 Determinations*

| Treatment | Cyclic AMP generated (pmol/mg protein/20 min) | P (Difference from basal) |
|---|---|---|
| Basal | 151±2 | — |
| $PGE_1$ 30 μM | 172±14 | NS |
| $PGE_2$ 30 μM | 141±17 | NS |
| PTH 2 μM | 162±18 | NS |
| Histamine 500 μM | 113±13 | NS |
| Vasopressin 200 mU/ml | 158±15 | NS |
| Gastrin 24 μM | 143±17 | NS |
| Glucagon 6 μM | 155±5 | NS |
| HGH 80 μM | 257±16 | <0·01 |
| Prolactin 80 μM | 232±6 | <0·001 |
| Isoprenaline 200 μM | 383±13 | <0·001 |
| SCT 3 μM | 832±88 | <0·001 |
| NaF 10 mM | 469±31 | <0·001 |

Fig. 1.—Adenylate cyclase activity in particulate preparations of BEN cells. Assay of adenylate cyclase activity was carried out, as described in Methods, in the presence of salmon calcitonin (●———●), porcine calcitonin (○———○), human calcitonin (▲———▲) or human calcitonin sulphoxide (□———□). Basal (unstimulated) adenylate cyclase activity (......) was $41 \pm 3$ pmol cyclic AMP generated/mg protein/20 min. Values are means ± s.e. of 3 determinations.

Fig. 2.—Binding of calcitonin to BEN cell membranes. [$^{125}$I]SCT was incubated with membranes, under conditions described in Methods, and bound separated from free labelled hormone after 15 min at 25°C. Competition for binding was tested with unlabelled salmon calcitonin (●), human calcitonin (○), and human calcitonin sulphoxide (▲). Values are means ± s.e. of 3 determinations.

[$^{125}$I]SCT bound to particulate preparations of BEN cells, and competition for binding was shown by unlabelled SCT and, at higher concentrations, by CT-M (Fig. 2). In much higher concentrations (3 $\mu$M), CT-M sulphoxide competed to some extent for binding. The concentration of SCT required for half-maximal inhibition of binding of labelled hormone was about 11 nM.

To determine whether hormone response varied with stages of culture confluence, the adenylate cyclase response was tested in membranes prepared from

TABLE II.—*Characteristics of the Adenylate Cyclase Response to Salmon Calcitonin (SCT) of BEN Cells at Various Times after Subculture. BEN Cells were Plated Out* ($3 \times 10^5$ *Cells/Flask) on Day* 0. *At Various Times Subsequently, Cells were Harvested, Particulate Preparations Obtained and the Adenylate Cyclase Response to SCT Determined.* **Maximum Response Occurred in the Presence of* 60 nM *SCT. Values in Columns* 2 *and* 3 *are Means* ± *s.e. from* 3 *Determinations*

| Day | Cyclic AMP (pmol/mg protein/20 min) Basal | Maximum* | Maximum/basal ratio | SCT concentration (nM) for half-maximal activation |
|---|---|---|---|---|
| 5 | 56±2 | 174±3 | 3·1 | 14·1 |
| 7 | 72±1 | 216±1 | 3·0 | 13·5 |
| 9 | 48±1 | 126±1 | 2·6 | 18·0 |
| 11 | 53±3 | 149±2 | 2·8 | 48·0 |
| 13 | 73±5 | 191±8 | 2·6 | 18·6 |
| 15 | 66±6 | 177±13 | 2·7 | 15·0 |

cultures at increasing time after subculture up to the time of confluence, which occurred at Day 15. The results (Table II) show that no change in responsiveness occurred. In view of the overall results of this experiment, the single observation (Day 11) of a possibly higher requirement of SCT for half the maximum activation of adenylate cyclase cannot be regarded as significant.

In view of the possible implications of the above observations, namely that a calcitonin-producing tumour cell could respond to its own apparent product, 2 further series of experiments were carried out. In the first, salmon calcitonin was applied to cultured cells for 4 h before the cells were washed and assayed for calcitonin-responsive adenylate cyclase activity. Whereas membranes from control cells responded as usual (Table III), those pre-incubated with calcitonin were found to be resistant to adenylate cyclase activation by the hormone. In the second, a sample of medullary thyroid carcinoma tissue was obtained at surgery from a patient known to have high levels of calcitonin. The adenylate cyclase response of membranes from this tumour was studied, and was found to be stimulated only by glucagon (Table IV). Salmon calcitonin was ineffective in the range from 60 nM to 6 μM.

TABLE III.—*Falcon Flasks of BEN Cells were Incubated for* 4 *h in Control Medium* (2 *Flasks) and Medium Containing* 150 nM *SCT* (2 *Flasks). After Thorough Washing of Cells, Particulate Preparations were Made and Assayed for Basal and Hormone-responsive Adenylate Cyclase. Values are Mean* ± *s.e. of* 3 *Determinations*

| | Adenylate cyclase activity (pmol cyclic AMP/incubation) Control | Pre-incubated 4 h with SCT (150 nM) |
|---|---|---|
| Basal | 15·2±1·1 | 18·3±0·7 |
| SCT 3 nM | 26·9±0·7 | 16·5±0·8 |
| SCT 30 nM | 29·2±1·1 | 17·6±0·4 |
| SCT 300 nM | 33·2±1·9 | 18·5±0·7 |
| CT-M 6 nM | 19·3±1·3 | 17·8±0·1 |
| CT-M 60 nM | 24·2±1·0 | 18·3±0·6 |
| CT-M 600 nM | 26·8±0·5 | 15·6±0·9 |

### DISCUSSION

The BEN cell line has been known for some time to produce CEA and calcitonin (Ellison *et al.*, 1975). The latter has been measured by radioimmunoassay, and has been shown recently to be secreted in a form which is of higher molecular weight than monomeric human calcitonin, although mild trypsin treatment converts it to the size of CT-M on gel filtration (Coombes *et al.*, 1977). The observation that BEN cells also had a calcitonin-responsive adenylate cyclase was a surprising one, but the present evidence

TABLE IV.—*Effect of Hormones on Adenylate Cyclase Activity of Particulate Preparations from Human Medullary Thyroid Carcinoma. Tumour was Removed at Surgery from a Patient with Elevated Plasma Calcitonin Levels. Within* 1 *h it was Homogenized in* 25 mM *Tris, pH* 7·4, *Centrifuged at* 2200 g *for* 15 *min, Resuspended and Assayed* (*see Methods*). *Means* ± *s.e. of* 3 *Determinations for Each Value. Prostaglandins* $E_1$, $E_2$ *and* $F_{2}\alpha$ *were Also Tested, and were Without Effect in Concentrations from* 1–100 μM

| Treatment | Concentration | Adenylate cyclase activity (pmol cyclic AMP/mg protein) |
|---|---|---|
| Control | — | 517±51 |
| Glucagon | 60 nM | 1154±65 |
| Glucagon | 600 nM | 1707±282 |
| Glucagon | 6 μM | 2087±155 |
| Salmon calcitonin | 60 nM | 630±35 |
| Salmon calcitonin | 600 nM | 552±50 |
| Salmon calcitonin | 6 μM | 573±6 |
| Isoprenaline | 6 μM | 868±112 |
| Isoprenaline | 60 μM | 938±60 |
| Gastrin | 2 μM | 525±14 |
| Gastrin | 20 μM | 517±26 |
| Sodium fluoride | 10 mM | 4609±396 |

confirms this in a number of ways. First, the various calcitonins had relative potencies in this system roughly paralleling their potencies in others, *e.g.* rat kidney and bone membranes (Marx, Woodard and Aurbach, 1972); second, the intact cells responded to hormone by an increase in intracellular cyclic AMP; and, finally, labelled calcitonin was found to bind to particulate cell preparations, while biologically active, unlabelled calcitonins competed for binding.

Although specific calcitonin receptors have been demonstrated in thymoma cell lines (Marx *et al.*, 1974), these were not associated with any demonstrable cell activation process. The BEN cells on the other hand possess a receptor which is probably concerned with the stimulation of adenylate cyclase by calcitonin. It is possible that the BEN cell population is not homogeneous, and contains one subpopulation which secretes calcitonin and one which has a calcitonin receptor. However, homogeneity, as assessed by morphological and functional (production of CEA and calcitonin) criteria, has been maintained over some 120 passages in 31 months (Ellison *et al.*, 1975; Ellison, unpub.). Alternatively, the cells might secrete calcitonin at one stage of their cycle and respond to it at another. Synchronized cultures would be necessary to exclude this possibility. Certain mouse neuroblastoma cell lines may provide a parallel for the observations reported here. The cells respond to nerve growth factor by increasing acetylcholinesterase production (Goldstein, Brodeur and Ross, 1973) and bind nerve growth factor at their membranes (Revoltella *et al.*, 1974). A mouse neuroblastoma cell line also secretes nerve growth factor (Murphy *et al.*, 1975), but the phenomena have not been studied concurrently.

The possibility therefore remains that the cells respond to their own hormonal product. Earlier studies had indicated that the BEN calcitonin was biologically active in one *in vitro* system, since it inhibited $^{45}$Ca release from mouse calvaria maintained in culture (Ellison *et al.*, 1975). Subsequent assays of partially purified media, however, have shown that the calcitonin from BEN cells is ineffective in lowering serum calcium in assay rats when given at doses (20 ng calcitonin immunoassay equivalents) which should be effective (Coombes *et al.*, 1977). Furthermore, the results of the experiments of Tables II and III favour the view that the calcitonin of BEN medium is not biologically active, because if it were it might be expected to induce a state of permanent " desensitization ", which would make it impossible to detect a calcitonin-responsive adenylate cyclase. This argument cannot be completely convincing, however, since it remains possible that the rate of calcitonin production (10 ng/confluent Falcon flask/day) is insufficient to induce resistance. Experiments to test this possibility are in progress. Finally, since trypsinization can convert BEN calcitonin to a form which may be identical with CT-M (Coombes *et al.*, 1977), the biological activity seen in bone culture may be explained by trypsin-like activity in the bone culture system.

The secretion of calcitonin by BEN cells is under a type of negative feedback control, in that progressive dilution of culture medium leads to a constant calcitonin concentration (Ellison *et al.*, 1976*a*,*b*). This is similar to the feedback shown for calcitonin release from medullary thyroid cancer cells (Ellison *et al.*, 1976*b*) and for thyroid C cells (Orme and Pento, 1976). The mechanism of this feedback is unknown and may be unrelated to the presence of the calcitonin receptor. Medullary thyroid carcinoma does not appear to possess calcitonin receptors linked to adenylate cyclase (Table IV). It should also be noted that in mammalian calcitonin-secreting cells, cyclic AMP stimulates hormone production rather than inhibits it (Bell and Queener, 1974; Gautvik and Tashjian, 1974). The mechanism could be different in BEN cells, although certain other features of the regulation of calcitonin secretion in culture are similar to those observed in mammalian C cells *in vitro* (Ellison *et al.*, 1976*a*).

Other possible autoregulatory roles for the calcitonin receptor are in the control of cell proliferation by means of calcitonin-induced changes in the cell's cyclic AMP, and in the control of secretion of CEA, also a product of BEN. $N^6$-monobutyryl cyclic AMP has been shown to enhance CEA production and increase the mean cell-doubling time in BEN cultures (Ellison *et al.*, 1976*b*). If BEN calcitonin does interact with calcitonin receptors on the cells, however, it seems likely that adenylate cyclase response would change with lengthening exposure to increasing hormone concentrations, since regulation of receptor numbers and affinities by hormones has been reported in a number of systems (Lesniak *et al.*, 1974; Hinkle and Tashjian, 1975; Mukherjee, Caron and Lefkowitz; 1976), and is suggested by the experiment described in Table III. Similarly, adenylate cyclase response would be expected to change with time after subculture, if the enzyme were associated with autoregulation. Table II shows, however, that the characteristics of the hormone-responsive adenylate cyclase did not change between 5 days after subculture and confluence (15 days).

Direct studies of the interaction of BEN calcitonin with BEN receptors have not been performed, because highly purified preparations of the hormone are not available. It is not possible at present to decide what functional relationship exists between the phenomena of human calcitonin production and responsiveness to calcitonin in these cancer cells.

This work has been supported by a grant from the Yorkshire Council of the Cancer Research Campaign. We are grateful to Professor A. M. Neville for helpful advice.

### REFERENCES

Bell, N. H. & Queener, S. (1974) Stimulation of Calcitonin Synthesis and Release *In vitro* by Calcium and Dibutyryl Cyclic AMP. *Nature, Lond.*, **248**, 343.

Boyd, H., Louis, C. J. & Martin, T. J. (1974) Activity and Hormone Responsiveness of Adenyl Cyclase during Induction of Tumours in Rat Liver with 3′-Methyl-4-dimethylaminoazo benzene. *Cancer Res.*, **34**, 1720.

Brown, B. L., Albano, J. D. M., Elkins, R. P. & Sgherzi, A. M. (1971) A Simple and Sensitive Saturation Assay Method for the Measurement of Adenosine 3′,5′-cyclic Monophosphate. *Biochem. J.*, **121**, 561.

Christoffersen, T. & Berg, T. (1975) Altered Hormone Control of Cyclic AMP Formation in Isolated Parenchymal Liver Cells from Rats Treated with 2-Acetylaminofluorene. *Biochim. biophys. Acta*, **381**, 72.

Christoffersen, T., Brønstad, G. O., Walstad, P. & Øye, I. (1974) Cyclic AMP Metabolism in Rat Liver during 2-Acetylaminofluorene Carcinogenesis. *Biochim. biophys. Acta*, **372**, 291.

Christoffersen, T., Morland, J., Osnes, J. B. & Elgio, K. (1972) Hepatic Adenyl Cyclase: Alterations in Hormone Response during Treatment with a Chemical Carcinogen. *Biochem. biophys. Acta*, **279**, 363.

Clark, J. F., Morris, H. P. & Weber, G. (1973) Cyclic Adenosine 3′,5′-Monophosphate Phosphodiesterase Activity in Normal, Differentiating, Regenerating and Neoplastic Liver. *Cancer Res.*, **33**, 356.

Coombes, R. C., Ellison, M. L., Easty, G. C., Hillyard, C. J., James, R., Galante, L., Girgis, S., Heywood, L., MacIntyre, I. & Neville, A. M. (1976) The Ectopic Secretion of Calcitonin by Lung and Breast Carcinomas. *Clin. Endocr.*, **5**, Suppl., 387s.

Coombes, R. C., Ellison, M. L., Girgis, S., Hillyard, C. J., MacIntyre, I. & Neville, A. M. (1977) The Heterogeneity of Human Calcitonin Secreted by Human Tumours *In vitro*. (Submitted.)

DeRubertis, F. R., Chayoth, R. & Field, J. B. (1976) The Content and Metabolism of Cyclic AMP and Cyclic GMP in Adenocarcinoma of the Human Colon. *J. clin. Invest.*, **57**, 641.

Ellison, M., Hillyard, C., Bloomfield, G. A., Rees, L. H., Coombes, R. C. & Neville, A. M. (1976*a*). Ectopic Hormone Production by Bronchial Carcinomas in Culture. *Clin. Endocr.*, **5**, Suppl., 397.

Ellison, M. L., Hillyard, C. J., Coombes, R. C. & Neville, A. M. (1976*b*) Control of Calcitonin Release *In vitro*. *J. Endocr.*, **71**, 85.

Ellison, M. L., Woodhouse, D., Hillyard, C. J., Dowsett, M., Coombes, R. C., Gilby, E. D., Greenberg, P. B. & Neville, A. M. (1975) Immunoreactive Calcitonin Production by Human Lung Carcinoma Cells in Culture. *Br. J. Cancer*, **32**, 373.

Gautvik, K. M. & Tashjian, A. H. (1974) Human Medullary Thyroid Carcinoma: Control of Calcitonin Secretion *In vivo* and in Tissue Culture. *Horm. Metab. Res.*, **6**, 70.

Goldstein, M. N., Brodeur, G. M. & Ross, D. (1973) The Effect of Nerve Growth Factor and Dibutyryl Cyclic AMP on Acetylcholinesterase in Human and Mouse Neuroblastomas. *Anat. Rec.*, **175**, 330.

Hartree, E. F. (1972) Determination of Protein: a Modification of the Lowry Method that gives a Linear Photometric Response. *Analyt. Biochem.*, **48**, 422.

Hinkle, P. M. & Tashjian, A. H. (1975) Thyrotropin-releasing Hormone Regulates the Number of its Own Receptors in the $GH_3$ Strain of Pituitary Cells in Culture. *Biochemistry*, **14**, 3845.

Hunt, N. H., Martin, T. J., Michelangeli, V. P. & Eisman, J. A. (1976) Effect of Guanylnucleotides on Parathyroid Hormone-responsive Adenylate Cyclase in Chick Kidney. *J. Endocr.*, **69**, 401.

Hunter, W. M. & Greenwood, F. C. (1962) Preparation of Iodine-131-labelled Growth Hormone of High Specific Activity. *Nature, Lond.*, **194**, 495.

Lesniak, M. A., Gorden, P., Roth, J. & Gavin, J. R. (1974) Binding of $^{125}$I-Human Growth Hormone to Specific Receptors in Human Cultured Lymphocytes. Characterization of the Interaction and a Sensitive Radio Receptor Assay. *J. biol. Chem.*, **249**, 1661.

Martin, T. J., Hunt, N. H., Boyd, H., Ellison, M. L., Michelangeli, V. P. & Atkins, D. (1976) Hormone Receptors and Cyclic Nucleotide Metabolism in Cancer Cells. *Clin. Endocr.*, **5**, Suppl., 373s.

Marx, S. J., Aurbach, G. D., Gavin, J. R. & Buell, D. W. (1974) Calcitonin Receptors on Cultured Human Lymphocytes. *J. biol. Chem.*, **249**, 6812.

Marx, S. J., Fedak, S. A. & Aurbach, G. D. (1972) Preparation and Characterization of a Hormone-responsive Renal Plasma Membrane Fraction. *J. biol. Chem.*, **247**, 6913.

Marx, S. J., Woodard, C. J. & Aurbach, G. D. (1972) Calcitonin Receptors of Kidney and Bone. *Science, N.Y.*, **178**, 99.

Minton, J. P., Wisenbaugh, T. & Matthews, R. H. (1974) Elevated Cyclic AMP Levels in Human Breast-cancer Tissue. *J. natn. Cancer Inst.*, **53**, 283.

Mukherjee, C., Caron, M. G. & Lefkowitz, R. J. (1976) Regulation of Adenylate Cyclase Coupled $\beta$-Adrenergic Receptors by $\beta$-Adrenergic Catecholamines. *Endocrinology*, **99**, 347.

Murphy, R. A., Pantazis, N. J., Arnason, B. G. W. & Young, M. (1975) Secretion of a Nerve Growth Factor by Mouse Neuroblastoma Cells in Culture. *Proc. natn. Acad. Sci. U.S.A.*, **72**, 1895.

Neher, R., Rimker, B., Maier, R., Byfield, P. G. H., Gudmundsson, T. V. & MacIntyre, I., (1968) Human Calcitonin. *Nature, Lond.*, **220**, 984.

Neville, A. M. & Cooper, E. H. (1976) Biochemical Monitoring of Cancer. *Ann. clin. Biochem.*, **13**, 283.

Orme, A. L. & Pento, J. T. (1976) Calcitonin-induced Inhibition of its Own Secretion. *Proc. Soc. exp. Biol. Med.*, **151**, 110.

Rees, L. H. & Ratcliffe, J. G. (1974) Ectopic. Hormone Production by Non-endocrine Tumours *Clin. Endocr.*, **3**, 263.

Revoltella, R., Bertolini, L., Pediconi, M. & Vignetti, E. (1974) Specific Binding of Nerve Growth Factor (NGF) by Murine C1300 Neuroblastoma Cells. *J. exp. Med.*, **140**, 437.

Ryan, W. L. & Heidrick, M. L. (1974) Role of Cyclic Nucleotides in Cancer. *Adv. Cyclic Nucleotide Res.*, **4**, 81.

Salomon, Y., Londos, C. & Rodbell, M. (1974) A Highly Sensitive Adenylate Cyclase Assay. *Analyt. Biochem.*, **58**, 541.

Schorr, I., Rathnam, P., Saxena, B. B. & Ney, R. L. (1971) Multiple Specific Hormone Receptors in the Adenylate Cyclase of an Adrenocortical Tumor. *J. biol. Chem.*, **246**, 5806.

Sraer, J. & Ardaillou, R. (1973) Renal Receptors of Salmon Calcitonin. In *Endocrinology* 1973, Ed. S. Taylor. London: Heinemann, p. 170.

Yalow, R. S. & Berson, S. A. (1966) Purification of $^{131}$I Parathyroid Hormone with Microfine Granules of Prepared Silica. *Nature, Lond.*, **212**, 357.


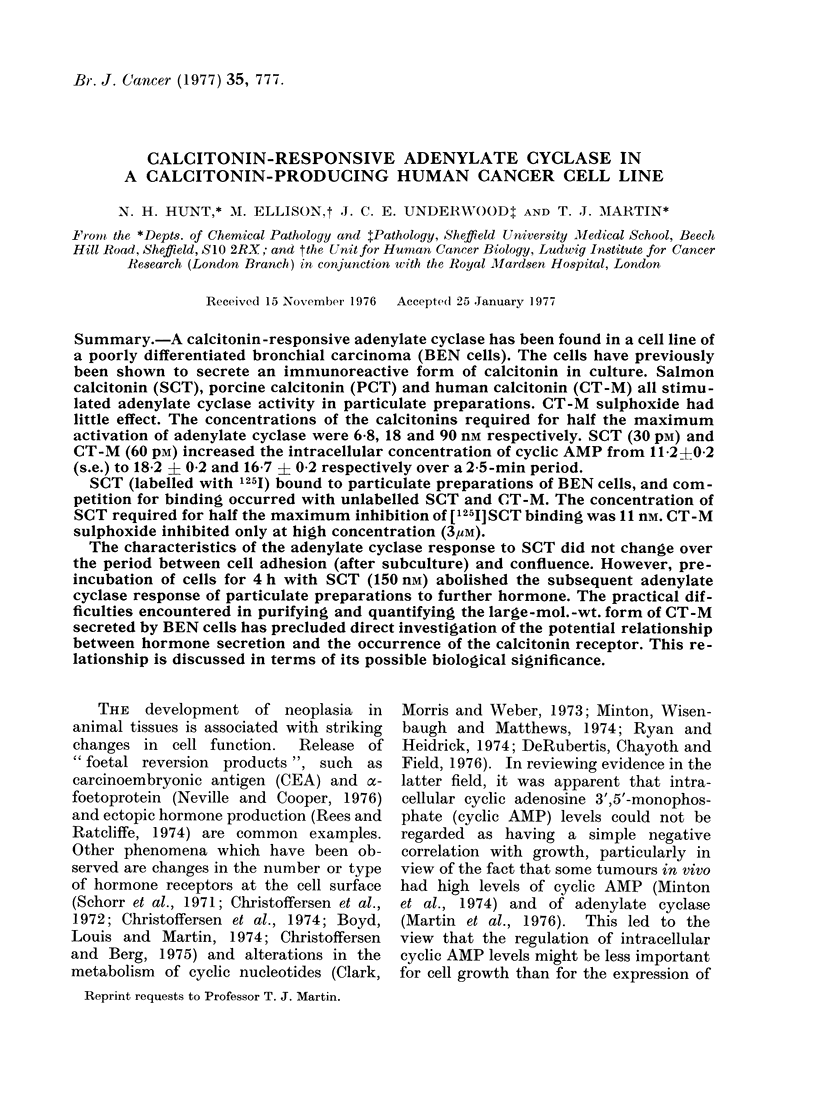

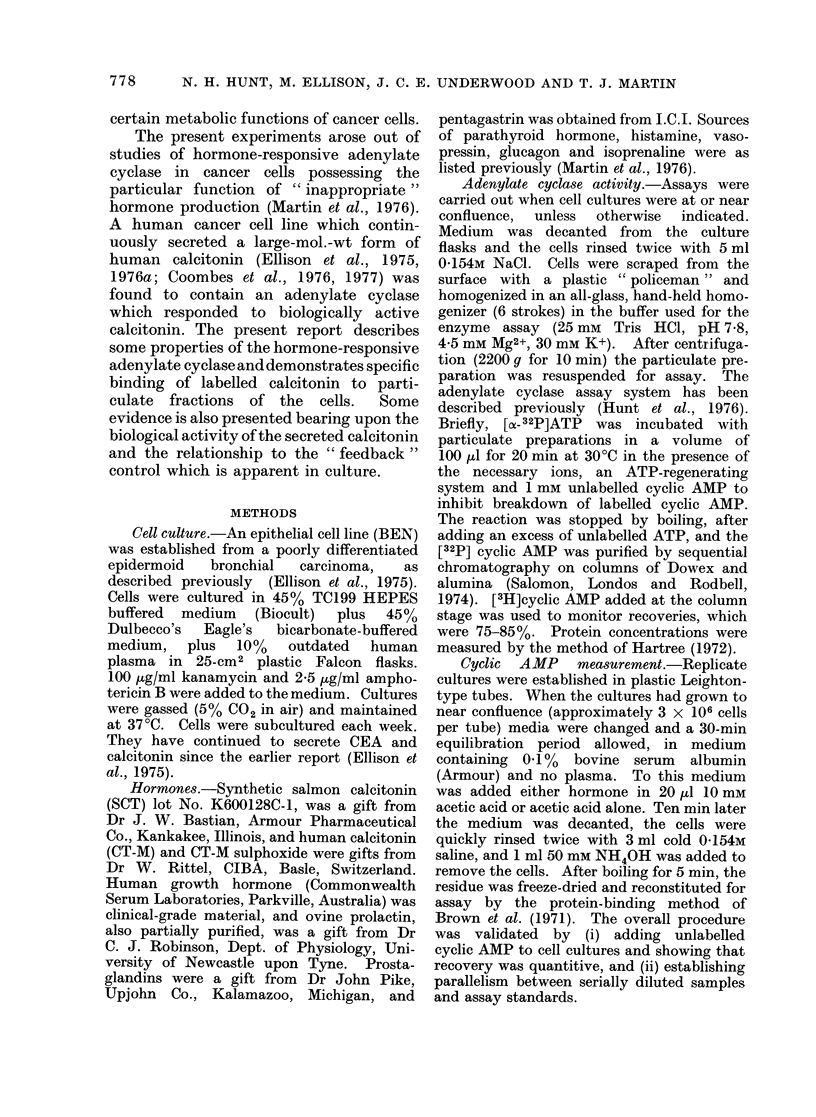

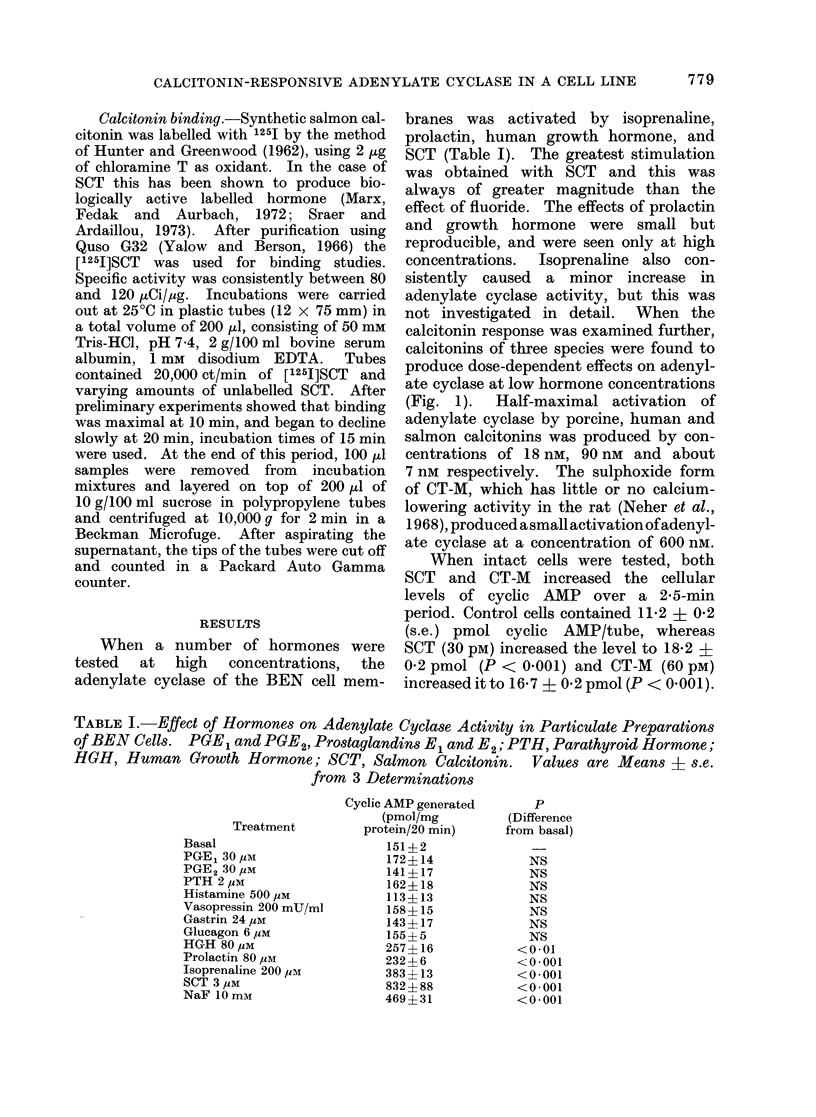

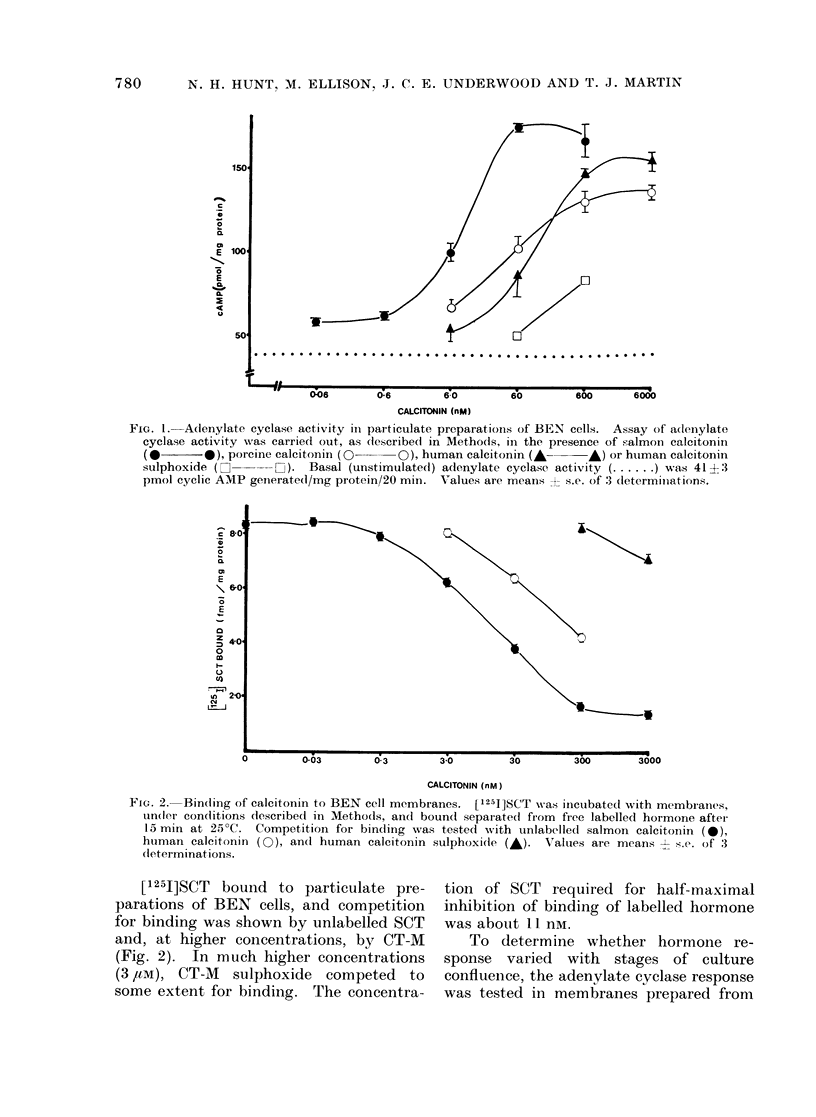

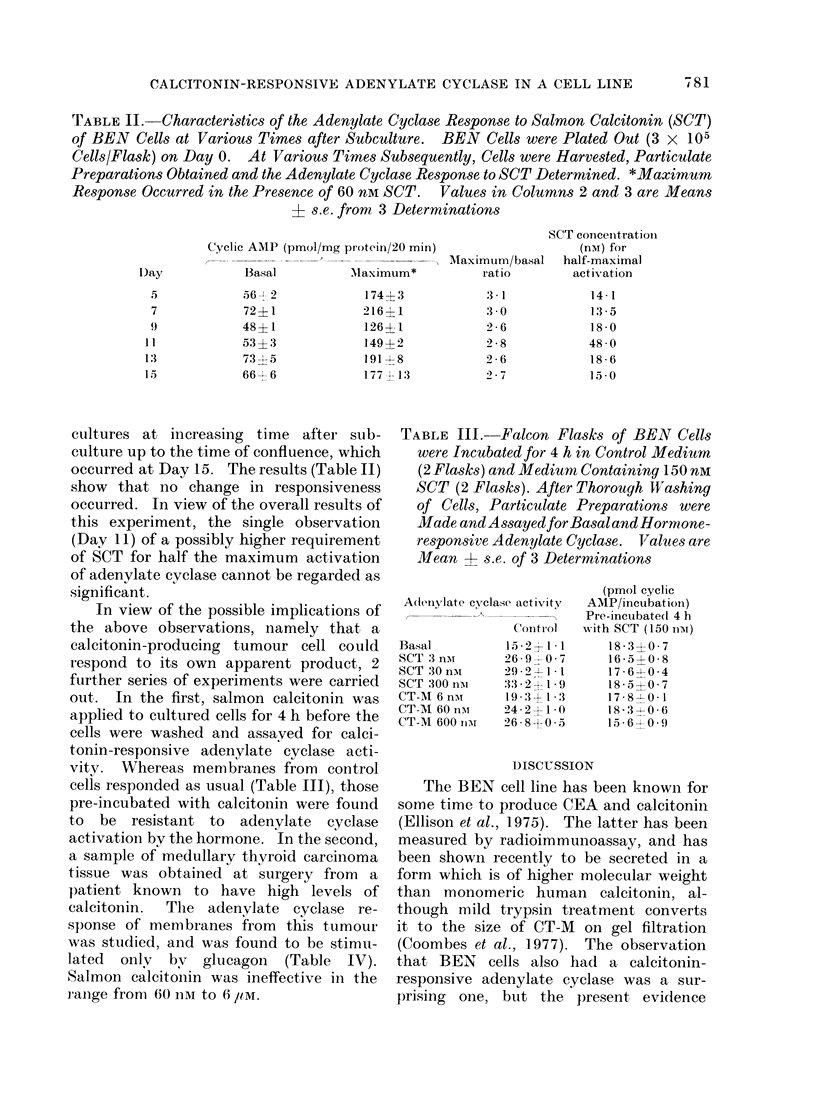

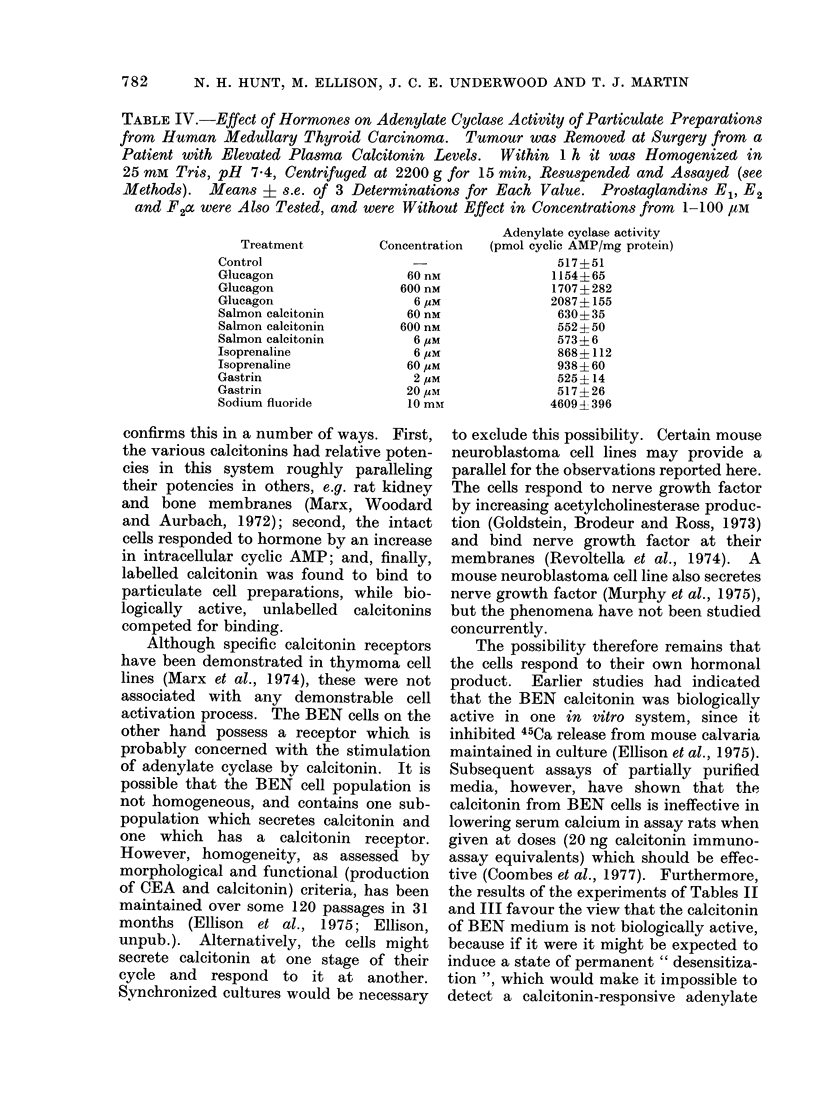

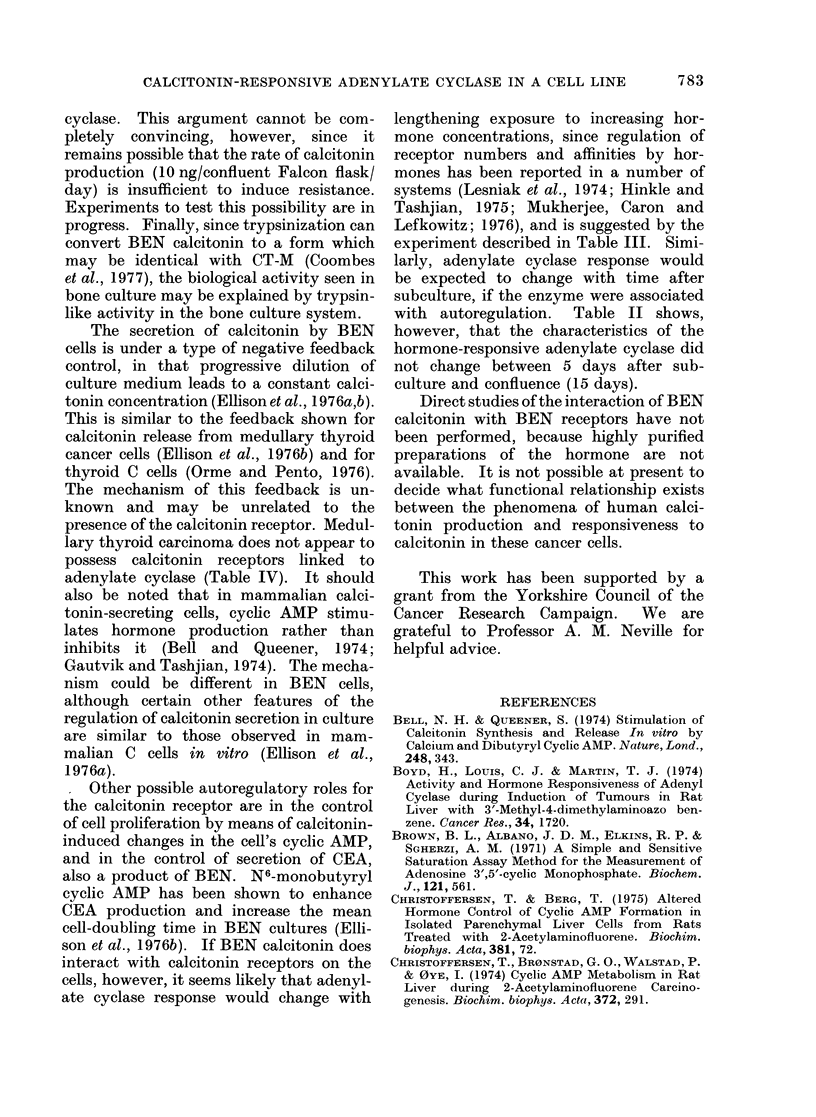

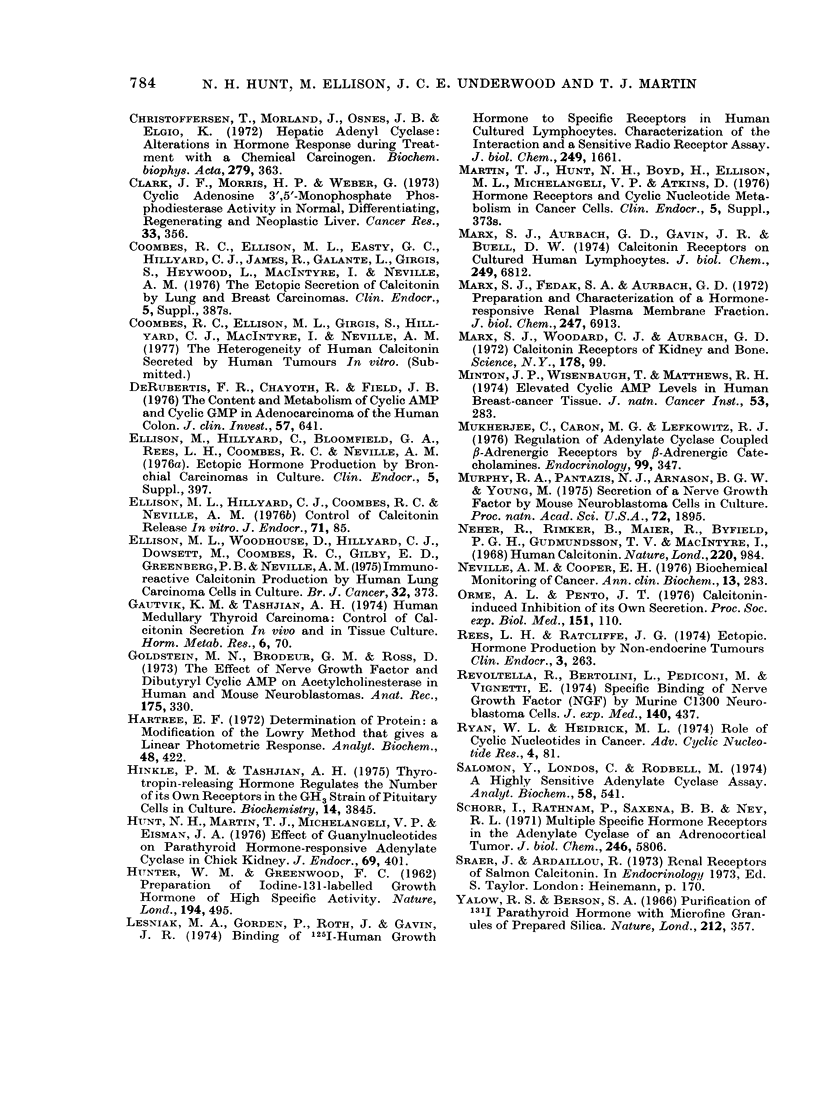


## References

[OCR_00810] Bell N. H., Queener S. (1974). Stimulation of calcitonin synthesis and release in vitro by calcium and dibutyryl cyclic AMP.. Nature.

[OCR_00816] Boyd H., Louis C. J., Martin T. J. (1974). Activity and hormone responsiveness of adenyl cyclase during induction of tumors in rat liver with 3'-methyl-4-dimethylaminoazobenzene.. Cancer Res.

[OCR_00823] Brown B. L., Albano J. D., Ekins R. P., Sgherzi A. M. (1971). A simple and sensitive saturation assay method for the measurement of adenosine 3':5'-cyclic monophosphate.. Biochem J.

[OCR_00830] Christoffersen T., Berg T. (1975). Altered hormone control of cyclic AMP formation in isolated parenchymal liver cells from rats treated with 2-acetylaminofluorene.. Biochim Biophys Acta.

[OCR_00845] Christoffersen T., Morland J., Osnes J. B., Elgjo K. (1972). Hepatic adenyl cyclase: alterations in hormone response during treatment with a chemical carcinogen.. Biochim Biophys Acta.

[OCR_00852] Clark J. F., Morris H. P., Weber G. (1973). Cyclic adenosine 3',5'-monophosphate phosphodiesterase activity in normal, differentiating, regenerating, and neoplastic liver.. Cancer Res.

[OCR_00859] Coombes R. C., Ellison M. L., Easty G. C., Hillyard C. J., James R., Galante L., Girgis S., Heywood L., MacIntyre I., Neville A. M. (1976). The ectopic secretion of calcitonin by lung and breast carcinomas.. Clin Endocrinol (Oxf).

[OCR_00892] Ellison M., Woodhouse D., Hillyard C., Dowsett M., Coombes R. C., Gilby E. D., Greenberg P. B., Neville A. M. (1975). Immunoreactive calcitonin production by human lung carcinoma cells in culture.. Br J Cancer.

[OCR_00929] HUNTER W. M., GREENWOOD F. C. (1962). Preparation of iodine-131 labelled human growth hormone of high specific activity.. Nature.

[OCR_00911] Hartree E. F. (1972). Determination of protein: a modification of the Lowry method that gives a linear photometric response.. Anal Biochem.

[OCR_00917] Hinkle P. M., Tashjian A. H. (1975). Thyrotropin-releasing hormone regulates the number of its own receptors in the GH3 strain of pituitary cells in culture.. Biochemistry.

[OCR_00923] Hunt N. H., Martin T. J., Michelangeli V. P., Eisman J. A. (1976). Effect of guanyl nucleotides on parathyroid hormone-responsive adenylate cyclase in chick kidney.. J Endocrinol.

[OCR_00935] Lesniak M. A., Gorden P., Roth J., Gavin J. R. (1974). Binding of 125I-human growth hormone to specific receptors in human cultured lymphocytes. Characterization of the interaction and a sensitive radioreceptor assay.. J Biol Chem.

[OCR_00944] Martin T. J., Hunt N. H., Boyd H., Ellison M., Michelangeli V. P., Atkins D. (1976). Hormone receptors and cyclic nucleotide metabolism in cancer cells.. Clin Endocrinol (Oxf).

[OCR_00951] Marx S. J., Aurbach G. D., Gavin J. R., Buell D. W. (1974). Calcitonin receptors on cultured human lymphocytes.. J Biol Chem.

[OCR_00957] Marx S. J., Fedak S. A., Aurbach G. D. (1972). Preparation and characterization of a hormone-responsive renal plasma membrane fraction.. J Biol Chem.

[OCR_00968] Minton J. P., Wisenbaugh T., Matthews R. H. (1974). Elevated cyclic AMP levels in human breast-cancer tissue.. J Natl Cancer Inst.

[OCR_00974] Mukherjee C., Caron M. G., Lefkowitz R. J. (1976). Regulation of adenylate cyclase coupled beta-adrenergic receptors by beta-adrenergic catecholamines.. Endocrinology.

[OCR_00980] Murphy R. A., Pantazis N. J., Arnason B. G., Young M. (1975). Secretion of a nerve growth factor by mouse neuroblastoma cells in culture.. Proc Natl Acad Sci U S A.

[OCR_00986] Neher R., Riniker B., Maier R., Byfield P. G., Gudmundsson T. V., MacIntyre I. (1968). Human calcitonin.. Nature.

[OCR_00990] Neville A. M., Cooper E. H. (1976). Biochemical monitoring of cancer. A review.. Ann Clin Biochem.

[OCR_00993] Orme A. L., Pento J. T. (1976). Evidence of calcitonin-induced inhibition of calcitonin secretion in porcine thyroid slices.. Proc Soc Exp Biol Med.

[OCR_00998] Rees L. H., Ratcliffe J. G. (1974). Ectopic hormone production by non-endocrine tumours.. Clin Endocrinol (Oxf).

[OCR_01003] Revoltella R., Bertolini L., Pediconi M., Vigneti E. (1974). Specific binding of nerve growth factor (NGF) by murine C 1300 neuroblastoma cells.. J Exp Med.

[OCR_01009] Ryan W. L., Heidrick M. L. (1974). Role of cyclic nucleotides in cancer.. Adv Cyclic Nucleotide Res.

[OCR_01014] Salomon Y., Londos C., Rodbell M. (1974). A highly sensitive adenylate cyclase assay.. Anal Biochem.

[OCR_01019] Schorr I., Rathnam P., Saxena B. B., Ney R. L. (1971). Multiple specific hormone receptors in the adenylate cyclase of an adrenocortical carcinoma.. J Biol Chem.

[OCR_01030] Yalow R. S., Berson S. A. (1966). Purification of 131-I parathyroid hormone with microfine granules of precipitated silica.. Nature.

